# Supplementary material for: Overexpression of pPLAIIIγ in Arabidopsis Reduced Xylem Lignification of Stem by Regulating Peroxidases
Source: Plants (Basel). 2022 Jan 13;11(2):200. doi: 10.3390/plants11020200 (PMC8777835; doi:10.3390/plants11020200)
Supplement: Supplementary file 1 [file plants-11-00200-s001.zip › plants-1534707-supplementary.pdf]

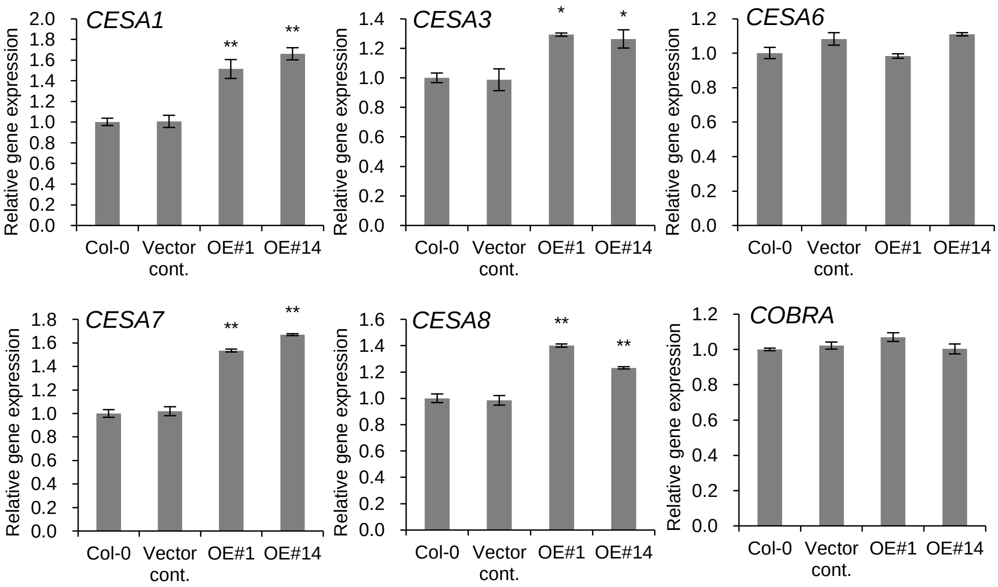

Figure S1. Genes involved in cellulose biosynthesis and production (*COBRA*) were upregulated in the stem of *pPLAIIIγ*OE lines. Each data point represents the average ± SE of three independent replicates at $P < 0.05$ (*) and $P < 0.01$ (**), respectively.

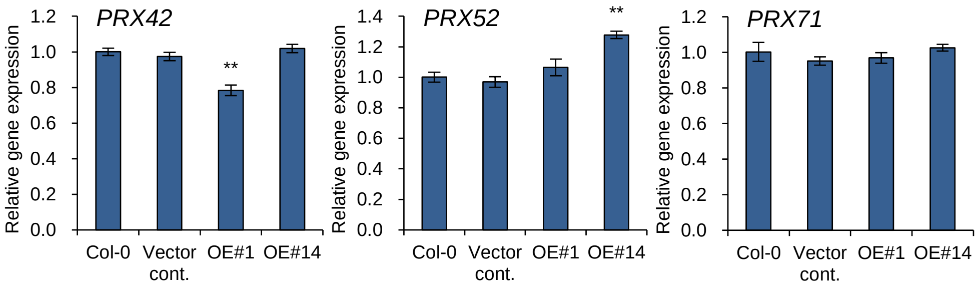

Figure S2. Expression levels of peroxidases in the stem of *pPLAIIIγ*OE lines.
Each data point represents the average ± SE of three independent replicates at P < 0.05 (*) and P < 0.01 (**), respectively.

Total RNA from 2-week-old seedling
(In figure 2A)

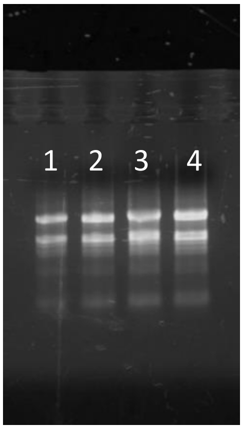

1: Col-0
2: Vector control (35S:YFP)
3: pPLAIIIγOE#5
4: pPLAIIIγOE#14

1% agarose gel, 100 V, 20 mins loading

Total RNA from 7-week-old stem
(In figure.3B, 4A, 4B, 5B, S1, S2)

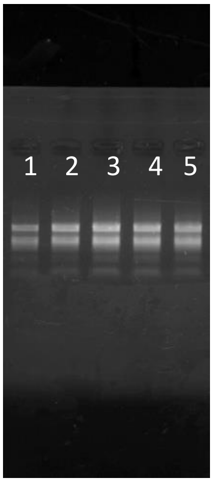

1: Col-0
2: Vector control (35S:YFP)
3: pPLAIIIγOE#1
4: pPLAIIIγOE#5
5: pPLAIIIγOE#14

1% agarose gel, 100 V, 20 mins loading

Figure S3. Agarose gel loading of total RNA used for qPCR. Total RNA isolated from 2- week-old seedling and 7-week-old stem was visualized in 1% of agarose gel
