# Supplementary material for: Overexpression of pPLAIIIγ in Arabidopsis Reduced Xylem Lignification of Stem by Regulating Peroxidases
Source: Plants (Basel). 2022 Jan 13;11(2):200. doi: 10.3390/plants11020200 (PMC8777835; doi:10.3390/plants11020200)
Supplement: Supplementary file 1 [file plants-11-00200-s001.zip › TABLE S1.pdf]

Table S1. List of DNA primers used for confirmation of gene insertion and qPCR.

Gene	Accession No.	Annotation	Primers used (5′-3′)
pPLAIIIα	At2g39220	pPLAIIIα-F<br>pPLAIIIα-R	GACGGATATGCAAGAACCGAGCAT<br>TTCGAACCTGACCCACCCGAACCA
pPLAIIIβ	At3g54950	pPLAIIIβ-F<br>pPLAIIIβ-R	GCG GCT TCC AAG TCG GCG AAT GAT<br>CTT AGC CGT CCC GGA ACA ACA ACC
pPLAIIIγ	At4g29800	pPLAIIIγ-F<br>pPLAIIIγ-R	TC GGT ACC GTC TAA AAG CTA ACG ATT<br>GG CCT AGG TCT ATC TTT AGA TAT GAG
pPLAIIIγ	At4g29800	pPLAIIIγ-KpnI-F<br>pPLAIIIγ-AvrII-R	GTC CAA CGT TAT CAA CGG CTC GAT<br>TCC TCC ACT TCC GCT CCC TGA TCT
pPLAIIIδ	At3g63200	pPLAIIIδ-F<br>pPLAIIIδ-R	ACA TTT TCA CAA AGC TTG AAC AGA<br>CCA AAA CCC TCT CCA TGC TCT TCC
β-actin	At5g09810	actin-2F<br>actin-2R	GTGTGTCTTGTCTTATCTGGTTCG<br>AATAGCTGCATTGTCACCCGATACT
MYB58	At1g16490	MYB58-F<br>MYB58-R	CCAGAGAACAGAGCTCTTCAAGAG<br>ATGTATGAGGAGCTCGTAACTCTC
MYB63	At1g79180	MYB63-F<br>MYB63-R	GAACAGCTCAGGCTCAAGAGCAAC<br>ATGTATCATGAGCTCGTAGTTCTT
PAL1	At2g37040	PAL1-F<br>PAL1-R	CAACGTACCCGTTGATTCAG<br>TCCTCGAAAGCTCCAATCTT
4CL	At1g51680	4CL-F<br>4CL-R	TCAACCCGGTGAGATTTGTA<br>TCGTCATCGATCAATCCAAT
HCT	At5g48930	HCT-F<br>HCT-R	CTCTTTCCAAAGCCCTTGTC<br>TCAGCCACAACGAAGAGAAC
COMT1	At5g54160	COMT1-F<br>COMT1-R	GTCGATTGCATTATGTTGGC<br>AGCCTGATGCTTTGGCTAAT
CCR1	At1g15950	CCR1-F<br>CCR1-R	TCCAGATGATCCGAAGAACA<br>CGCCTTAAGAGCCTCGTAGT
F5H2	At5g04330	F5H2-F<br>F5H2-R	ATCATGGATGTGATGTTCGG<br>ATCTCGGTTAGCACCCATTC
CESA1	At4g32410	CESA1-F<br>CESA1-R	CCC ATG GCC ATA CGG TTT CT<br>ACA GGT TGC CGT GGA TCA AT
CESA3	At5g05170	CESA3-F<br>CESA3-R	TAC TCG TGG GAA GGG AGA GG<br>CAG AGA GGC GTT CAG GTG AG
CDSA6	At5g64740	CESA6-F<br>CESA6-R	TTC ACT TGG TGG TCA TGG CA<br>ACC ATA GGC CTT GGA TGT GC
CESA7	At5g17420	CESA7-F<br>CESA7-R	GTTTTGCGTTGGGCACTTG<br>AGTTTGCCTCCTTTGTAGCCATA
CESA8	At4g18780	CESA8-F<br>CESA8-R	TCTTTGTGGCTTGTCATGAGTGT<br>CAACGCAAGCAAATTCTTCGA
COBRA	At5g60920	COBRA-F<br>COBRA-R	GACAATTGCGTCATGCCTCC<br>GGAACCACCGTTGGGAAGAA
PRX42	At4g21960	PRX42-F<br>PRX42-R	AAGCAGGACGGATATGCTCG<br>AGTGAGTTCTTCCCACGCTG
PRX52	At5g05340	PRX52-F<br>PRX52-R	GCTTTGTCAACGGATGCGAC<br>CTGAATTGCGGTTTGGAGCC
PRX64	At5g42180	PRX64-F<br>PRX64-R	GGAGGTTGACCCAACACTAAA<br>TCACGGTTCCATCCATGTTC
PRX71	At5g64120	PRX71-F<br>PRX71-R	GGGACGTAGAGATGGTAGAGTT<br>GACGACGAGATCACGAGTATTG
PRX72	At5g66390	PRX72-F<br>PRX72-R	TCA GCG AGA AAC GAT CAA AC<br>CCA AGA TAT CAG CGC AAG AA
